# The talking heads attentional bias assessment task: A readily available, reliable, and effective task for assessing attentional bias

**DOI:** 10.3758/s13428-026-03045-6

**Published:** 2026-05-22

**Authors:** Mahdi Mazidi, Colin MacLeod, Seyran Ranjbar, Owen Myles, Ben Grafton

**Affiliations:** 1 Centre for the Advancement of Research on Emotion, University of Western Australia, Perth, WA Australia; 2 The Kids Research Institute Australia, Perth, WA Australia; 3 School of Psychological Science M304, The University of Western Australia, 35 Stirling Highway, Crawley, 6009 Australia

**Keywords:** Attentional bias, Internal consistency, Anxiety, Attentional probe

## Abstract

Cognitive theories contend that attentional bias to negative information contributes to elevated trait anxiety. However, research in this area has been hindered by the lack of a standardized assessment task that demonstrates the required qualities, including strong internal consistency and ecological validity. The present study aimed to develop and validate the Talking Heads Attentional Bias Assessment Task, an easy-to-implement measure that overcomes the limitations of previous attentional bias assessment tasks. The task employs video-based stimuli conveying emotionally negative or benign information about issues individuals commonly encounter in their daily lives and uses a dual-probe methodology to assess attentional bias. A sample of 168 undergraduate students completed the task along with measures of trait and state anxiety. The results demonstrated that the attentional bias index derived from the task showed excellent internal consistency across multiple reliability assessment methods. Greater attentional bias to negative information was also significantly associated with greater trait anxiety, confirming the task’s sensitivity to anxiety-linked individual differences in such attentional bias. Furthermore, mediation analyses revealed that attentional bias mediated the association between trait anxiety and state anxiety elevation in response to emotional information. We discuss the study’s important theoretical and methodological implications, and convey the task’s capacity to enhance assessment of attentional bias across a wide range of psychological domains.

**Supplementary Information:**

The online version contains supplementary material available at 10.3758/s13428-026-03045-6.

## Introduction

Across recent years, theoretical models spanning various clinically relevant conditions, such as anxiety dysfunction, have implicated biased attention to relevant types of information in the development and maintenance of these conditions (Bollen et al., 2022; De Houwer & Koster, 2023; Todd et al., 2015; Van Bockstaele et al., 2014). Cognitive theories propose that an attentional bias toward negative information plays a key role in the development and maintenance of elevated trait anxiety (Kabacoff et al., 1997; Myles et al., 2020). The existence of this proposed attentional bias has been supported by numerous studies (Beck et al., 1985; Cisler & Koster, 2010; Williams et al., 1997). Specifically, such studies have shown that compared to people with lower trait anxiety, individuals with elevated trait anxiety show greater attention to emotionally negative information (information that is emotionally aversive and/or unpleasant) relative to emotionally benign information (information that is either emotionally neutral or positive) (Bar-Haim et al., 2007; Clauss et al., 2022).

However, progress in this research area has been hindered by the lack of a standardized task that assesses attentional bias to generally negative information and genuine issues that individuals commonly encounter in real life. Such a task should be easily deployable, should be readily adaptable, and should demonstrate several important qualities, including ecological validity, internal consistency, and sensitivity to the phenomenon of interest. The purpose of this study is to develop, validate, and make available to fellow researchers an easy-to-deploy task that demonstrates these qualities. This introduction will review the most widely used approach to assessing attentional bias, discuss its limitations, identify three important qualities that an appropriate attentional bias assessment task should demonstrate, and outline how the new task is designed to demonstrate these qualities.

To date, the most commonly used approach to assess attentional bias has been the attentional probe task (MacLeod et al., 1986). In the conventional version of this task, participants are briefly presented with pairs of stimuli, either words or still images, that each consist of one negative and one benign member. To measure how participants distribute their attention between these stimuli, a single visual probe is subsequently presented in the location where either the negative or benign stimulus had just been shown. Participants are required to quickly identify this probe, which remains visible until they respond. Relative speeding of their response to probes that appear where the negative stimulus was presented, rather than the benign stimulus, provides an index of attentional bias towards negative information.

While this conventional probe assessment approach has been widely used, it has three limitations that the present task variant is designed to overcome, namely low ecological validity, poor internal consistency, and, consequently, suboptimal sensitivity to the phenomenon of interest. Each of these limitations, and how the novel task is designed to overcome them, will be considered in turn. Ecological validity refers to how much the assessment task resembles and reflects the relevant characteristics of the phenomenon of interest in the real world (Osborne-Crowley, 2020; Tupper & Cicerone, 1990). Low ecological validity reduces the generalizability of laboratory findings to everyday life and diminishes their predictive power for real-world functioning (Chaytor & Schmitter-Edgecombe, 2003; Richards et al., 2014). The conventional attentional probe tasks have been criticized for their low ecological validity, primarily because this assessment approach involves the presentation of informationally impoverished stimuli, including single words or still images (MacLeod et al., 2019). Such stimuli are far less rich than the types of emotional information that people commonly process in real life, which is complex, dynamic, and continuous (Lazarov et al., 2016; Richards et al., 2014; Soleymani et al., 2022). Therefore, a quality that an appropriate attentional bias assessment task should demonstrate is the use of stimuli that more closely reflect the complexity of the emotional content people encounter in everyday life, such as a news bulletin reporting bad versus good news (Grafton et al., 2021). In contrast to words and still images, video-based stimuli are not only richer in content but also permit the delivery of complex, dynamic, and continuous information. Employing such stimuli in an attentional bias assessment task would therefore improve ecological validity (Risko et al., 2012). Accordingly, we implemented videos as stimuli in the current study. Specifically, we utilized video clips featuring individuals conveying emotionally negative or benign information relevant to a broad range of everyday situations. To achieve this, we used dual videos featuring head-and-shoulders displays of individuals presenting negative and benign perspectives on a wide variety of topics. To verify the ecological validity of the video stimuli, we asked an independent sample of participants to categorize the videos according to whether they addressed genuine issues people commonly encounter in their lives. To enable other researchers to adapt the assessment task to their own needs readily, we used AI-generated talking people in these videos (rather than real actors), as this readily allows our stimuli to be adapted to present information in other languages or with amended content.

Another quality that an appropriate attentional bias assessment task should demonstrate is strong internal consistency. Conventionally, an internal consistency coefficient below 0.7 is considered to be low, while coefficients of 0.70 are considered acceptable, coefficients of 0.80 are considered good, and coefficients of 0.90 or higher are considered excellent (Green et al., 2016; Groth-Marnat & Wright, 2016). It is now widely recognized that the attentional bias index yielded by the conventional attentional probe task has low internal consistency. For example, Schmukle (2005) and Staugaard (2009) reported internal consistency coefficients of 0.22 and 0.27, respectively, and more recent studies have confirmed this issue in the conventional attentional probe task (e.g., Kappenman et al., 2014; Mazidi et al., 2019; Price et al., 2015; Van Bockstaele et al., 2020; Xu et al., 2024). Group differences can potentially be revealed by instruments with relatively low levels of internal consistency (MacLeod et al., 2019; van Rooijen et al., 2017), which may explain the relative robustness of the finding that cohorts of high-trait-anxious and low-trait-anxious participants significantly differ from each other in terms of the attentional bias index provided by the conventional dot probe assessment task. However, assessment instruments with low internal consistency perform notoriously poorly when employed to index effects shown by individuals, as is required for many purposes including the assessment of change at the level of individuals, or the investigation of mediation effects (McNally, 2019; Rodebaugh et al., 2016). Thus, to be of maximum utility, an attentional bias assessment task must yield an index of attentional bias that has minimally acceptable and ideally excellent internal consistency. Unless this quality is shown, the assessment instrument will be of limited value in research that seeks to advance understanding of anxiety-linked attentional bias.

In response to calls to develop a measure of attentional bias with acceptable internal consistency, Grafton et al. (2021) recently introduced the dual-probe attentional bias assessment task. This new approach differs in some important ways from conventional attentional probe tasks. Rather than presenting a *single probe* in one of the two pertinent screen locations that remains on screen until identified, the dual-probe task instead presents *probe pairs* very briefly (200 ms), with one member of each probe pair appearing in each of the two pertinent screen locations. Participants are instructed to identify whatever probes they see. Due to the very brief presentation of probe pairs, participants can see only one of the two probes, and it is assumed that this is more likely to be the probe at the screen location they are attending to. Therefore, attentional distribution is inferred from the relative frequency with which participants accurately identify probes in each of the two locations. Specifically, an index of attentional bias to negative information is obtained by calculating the proportion of correctly identified probes that had been presented at the location of negative stimuli. When a higher proportion of accurately identified probes appear at the location of negative stimuli rather than at the location of benign stimuli, this indicates an attentional bias toward negative information. Using this novel dual-probe task, Grafton et al. (2021) found the dual-probe attentional bias assessment task to yield an attentional bias index with internal consistency of 0.97. Therefore, the current study employed the dual-probe approach to maximize the likelihood of achieving high internal consistency in the attentional bias assessment task. The criterion for the internal consistency will be met if the measure of attentional bias yielded by the present attentional bias assessment task demonstrates an internal consistency coefficient of at least 0.70, though ideally, this coefficient would be above 0.90.

A third quality that an appropriate assessment task intended to measure anxiety-linked attentional bias should meet is that the attentional bias index it yields must demonstrate sensitivity to the previously established trait anxiety-linked attentional bias toward negative information. This trait-anxiety-linked attentional bias effect is so well-established that meta-analytic evidence indicates it would require over 10,000 consecutive studies with null results to reduce the effect to non-significance (Bar-Haim et al., 2007). The criterion for establishing such a sensitivity to anxiety-linked individual differences will be the presence of a significant positive correlation between the attentional bias index and trait anxiety scores. To determine whether the present attentional bias assessment task meets this criterion, we examined whether variation in trait anxiety was indeed associated with the resulting measure of attentional bias to negative information. In addition to the assessment of attentional bias and trait anxiety, we also assessed state anxiety at key junctures of the study to compute the degree to which exposure to emotional information conveyed by our stimulus videos elevates state anxiety. Exploratory analyses were conducted to examine whether changes in state anxiety from before to after the presentation of emotional information were predicted by attentional bias toward negative information. Moreover, it is plausible that the extent to which individuals with higher trait anxiety exhibit greater attentional bias toward negative information may determine whether these individuals show larger elevations in state anxiety following the presentation of emotional information. Therefore, we took the opportunity to test a mediation model to determine whether the attentional bias index to negative information, yielded by the present assessment task, mediates the association between trait anxiety and the observed elevation in state anxiety in response to emotional information.

In summary, the present study was designed to develop and validate a new, easy-to-implement Talking Heads Attentional Bias Assessment Task that employs ecologically valid emotional information and is intended to yield an attentional bias measure with good internal consistency that is significantly associated with the anxiety-linked measures of interest.

## Method

### Participants

In total, 168 first-year undergraduate students from the University of Western Australia (125 females) participated in the study. This sample size substantially exceeded the sample sizes of all prior studies using the dual-probe paradigm (Grafton et al., 2021, 2023; Rudaizky et al., 2026; Todd et al., 2022; Wiechert et al., 2021), and was significantly larger than the typical samples reported in attentional bias research, which average fewer than 50 participants per study (Bar-Haim et al., 2007). Participants’ ages ranged from 17 to 42 years (*M* = 19.38, *SD* = 4.21). In terms of ethnicity, 112 identified as White (66.67%), 50 as Asian (29.76%), one as Native Hawaiian or Other Pacific Islander (0.6%), two as African American (1.19%), and three as having a mixed ethnic background (1.78%). Participant recruitment aimed to obtain a sample that varied normally in trait anxiety. This aim was achieved, as the trait anxiety scores demonstrated a normal distribution with skewness (– 0.04) and kurtosis (– 0.55) both below 1, and scores ranging from 23 to 73 (*M* = 46.69, *SD* = 11.08). Students received partial course credit for their participation.

### Materials

#### Questionnaire measures

##### Assessment of individual differences in trait anxiety

The Spielberger State-Trait Anxiety Inventory (STAI-T; Spielberger et al., 1983) was employed to assess trait anxiety. The STAI-T is a widely used measure of trait anxiety and has good reliability and validity (Barnes et al., 2002; Oei et al., 1990). Possible sum scores ranged from 20 to 80, where greater scores indicated greater trait anxiety. The internal consistency of the scale was excellent in the current study (Cronbach’s α =.94).

##### Assessment of state anxiety

The assessment of state anxiety was required to measure the impact of exposure to emotional information on state anxiety. State anxiety was measured using the short form of the STAI (STAI-S6; Marteau & Bekker, 1992), which consists of three anxiety-present items (e.g., “*I am tense*”) and three anxiety-absent items (e.g., “*I am relaxed*”). The STAI-S6 has been shown to be a valid and reliable measure of state anxiety and is sensitive to changes in state anxiety (Marteau & Bekker, 1992; Tluczek et al., 2009). Participants responded to each item on a continuous scale ranging from Not at all (0) to Very much (100). In the current study, the internal consistency of this scale was high, with Cronbach’s alpha ranging from .88 to .91.

#### Stimulus materials

The present task required the simultaneous presentation of video pairs, with one member conveying negative information and the other conveying benign information about genuine issues people may commonly encounter in real life. The following three subsections will first describe the informational content of the talking head videos as scripted, then how the talking head videos were created, and, finally, how they were combined to form the dual videos used in the attentional bias assessment task.

##### Emotional information scripts

The research team created 48 scripts, each conveying either a negative or a benign perspective on one of six topics concerning genuine issues people may commonly encounter in real life. These six topics were security issues (e.g., rise in crime rates vs. low crime rates), health issues (e.g., unhealthy food additives vs. healthier food improvements), appearance issues (e.g., fear of being judged for weight vs. acceptance of different body sizes), social issues (e.g., anxiety about party hosting vs. enthusiasm for social gatherings), employment issues (e.g., anxiety about performance reviews vs. benign experiences from performance reviews), and safety issues (e.g., concern about building fire risks vs. enhanced fire prevention measures). Eight scripts were generated for each topic. Four of the scripts concerning each topic conveyed negative information, and four conveyed benign information. The emotional valence of each script (negative or benign) was clearly conveyed from the first sentence. The scripts were intended to be spoken in approximately 60 s, and so each script was 200 words long. To verify that the scripts addressed genuine issues that people commonly engage with in real life and that the scripts aligned with their intended valence, ten students categorized each script based on these two criteria: (1) whether the perspective described in the script concerned a genuine real-life issue and (2) its emotional valence. The results confirmed that the scripts met both criteria, with an average agreement of 97% (*SD* = 0.05) for scripts describing a genuine real-life issue. Categorization of emotional valence demonstrated 100% alignment with the intended valence; all intended negative scripts were classified as emotionally negative, and all intended benign scripts were classified as emotionally benign.

##### Talking head videos

We used AI technology to create the talking head videos, and in each such talking head video, one script was read out by a (simulated) person. The use of AI-generated videos of this nature enables the current task to be readily adapted by other researchers, to suit their research questions and participant populations (for example, by easily changing the informational content that is spoken, or by altering the language of the talking head). The AI-based faces were generated using the FaceGenerator platform (https://generated.photos/face-generator). The faces that were to speak the negative scripts were generated with a mildly frightened expression, whereas those that were to speak the benign scripts were generated with a mildly happy expression. A total of 48 such faces were generated, evenly split between male and female. To create the talking-head videos of these faces speaking, we submitted each face to the D-ID platform (https://www.d-id.com/), which generated videos of that face speaking one of the 48 scripts. Male faces spoke in male voices while female faces spoke in female voices, and we ensured that every voice in each gender was unique using voice-altering software (‘MorphVOX Pro’ and ‘Adobe Audition’). Each talking head video was edited to be exactly 60 s in length and measured 13.4 × 13.4 cm on the 22-inch monitors used for data collection in this study. To verify that the final multi-modal video stimuli conveyed the intended emotional tone (i.e., that negative videos were perceived as negative and benign videos as benign), we conducted a validation check on the final videos. Ten independent participants viewed each video and rated its overall emotional tone (negative vs. benign) and the perceived facial expression of the talking head on a scale ranging from happy to frightened. The results demonstrated full alignment between intended and perceived emotional valence: all intended negative videos were judged as emotionally negative, and all intended benign videos were judged as emotionally benign. Ratings of facial expression (happy vs. frightened) also showed the expected pattern, confirming that the talking heads’ expressions were perceived as consistent with the intended emotional tone.

##### Dual video creation

Our attentional bias assessment task presented participants with dual videos, each containing a pair of talking head videos. In each of these dual videos, one talking head was male, and the other was female, and the two differed in whether they conveyed negative or benign information about the same general topic. To create the required stimuli, the 48 talking-head videos were paired to produce 24 dual videos. In half of the dual videos, the negative information was conveyed by the male talking head and the benign information was conveyed by the female talking head, while in the other half this was reversed. The voice of each talking head in the dual videos was delivered through the corresponding side of the headphones (i.e., the voice from the talking head on the right side of the screen came through the right side of the headphones, and the voice from the talking head on the left side of the screen came through the left side). The center of each talking head video was positioned at the center of its respective half of the screen.

In keeping with Grafton et al., (2021) within each dual video, on nine pseudorandomized occasions (every 5, 6, or 7 s), the two talking head videos switched positions on screen, and their associated audio channels were also switched accordingly. Thus, during each 60-s dual video, the positions of the talking head videos and their corresponding audio channels switched nine times, for a total of ten video arrangements per video pair. Across the 24 dual videos, 12 started with the male talking head on the left and 12 started with the female on the left, and for each of these subsets of dual videos, half started with the negative content being conveyed by the initially left talking head, while half started with the benign content being conveyed by the initially left talking head. An example dual video can be found here: https://youtu.be/V8jTTKMMNrg.

#### Talking heads attentional bias assessment task

**Fig. 1:**
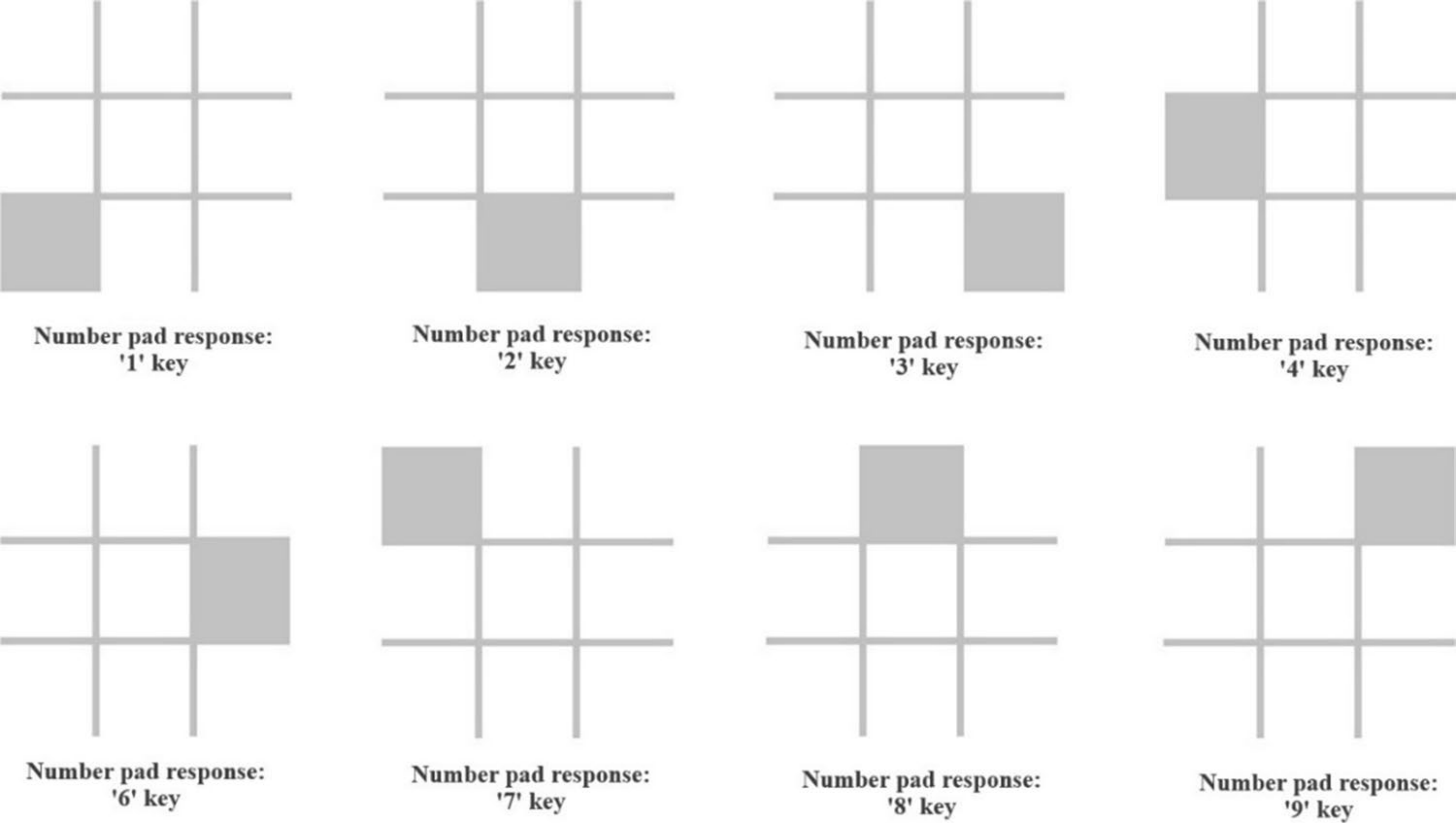
Probe stimuli presented in the dual-probe task and corresponding number pad response

**Fig. 2:**
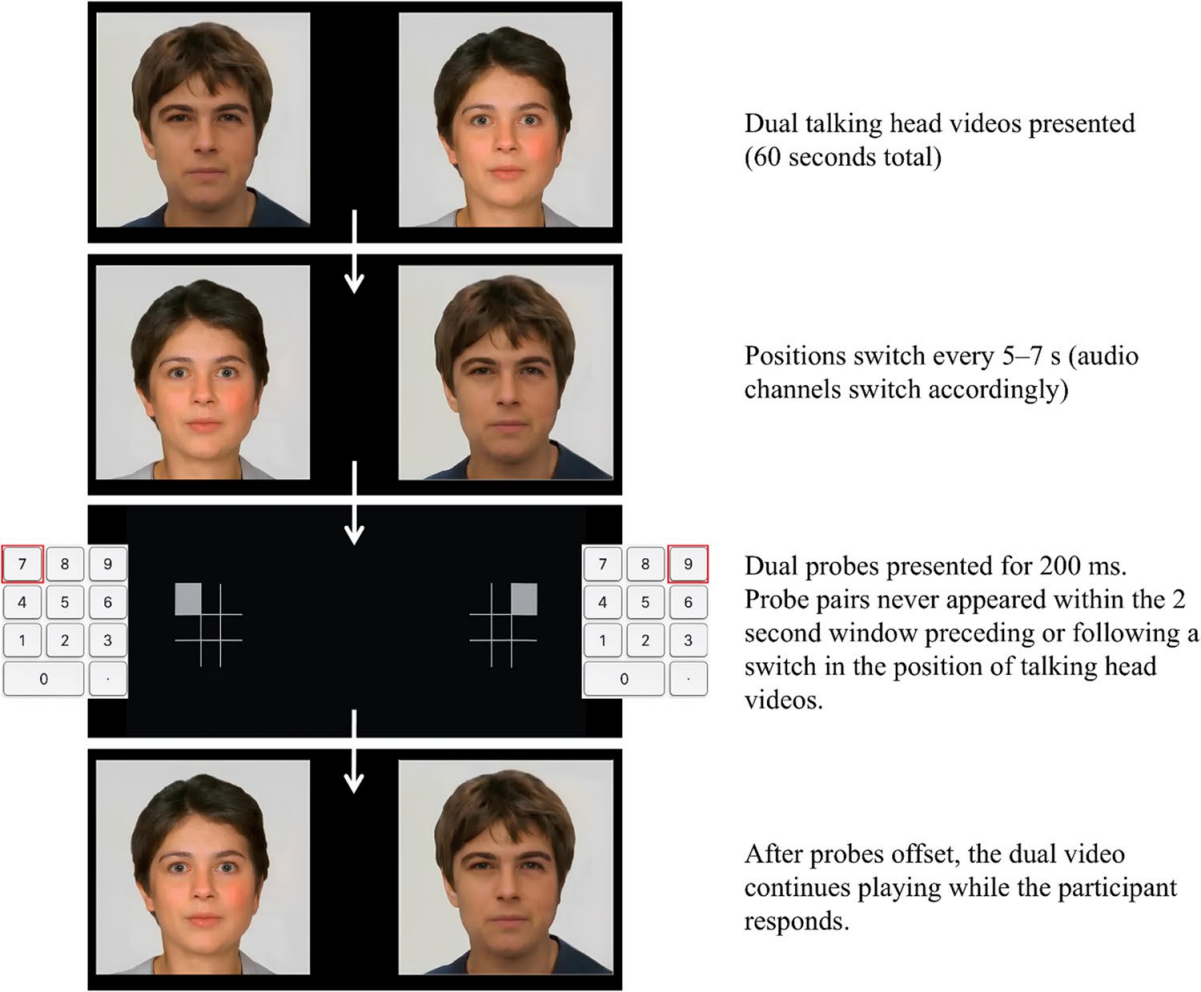
Example trial from the Attentional Bias Assessment Task. The probes are significantly enlarged for display purposes. Correct responses are outlined in *red*

To assess attentional bias to negative information, the dual-video format was used to create the Talking Heads Attentional Bias Assessment Task. In this task, we adopted the dual-probe approach developed by Grafton et al. (2021) to assess the extent to which participants showed an attentional bias toward the talking head in each dual video that conveyed negative rather than benign information. Specifically, during the presentation of each dual video, at ten pseudorandomized intervals (once per dual-video arrangement), two probes were simultaneously presented for just 200 ms. Each probe consisted of a grey 3 × 3 grid on a black background, measuring 2.2 × 2.2 cm on the screen, with one of the outer eight grid positions occupied by a small grey square (see Fig. 1). One probe appeared in the center of the location in which the left talking head video was playing, and the other probe appeared in the center of the location in which the right talking head video was playing. The identities of the two probes always differed, though participants were informed that the identities of the two probes in a pair were always the same. Participants were required to indicate the identity of any probes they saw by pressing the key on the 3 × 3 number pad corresponding to the location of the grey square within the 3 × 3 grid of the probe. After the probe offset, the dual video resumed playing while participants responded to the probe (please see Fig. 2 for an example trial).

As Grafton et al. (2023) point out, attentional distribution can be accurately inferred only if participants correctly identify probes. Thus, in keeping with Grafton et al., participants who failed to identify probes within at least 80% of dual-probe presentations were excluded. The rationale underpinning the dual-probe approach is that participants will be more likely to see the briefly presented probes at the location of the talking head they are attending to. Therefore, attentional bias to negative information is indexed by the degree to which participants are relatively more likely to accurately identify those members of probe pairs that appear in the locus of the negative talking head videos, rather than in the locus of the benign talking head videos. Therefore, for each participant, we obtained an index of their attentional bias to negative information by computing the proportion of the probes they accurately identified that had appeared in the locus of the negative talking head videos, using the following equation:

$$\mathrm{Index}\;\mathrm{of}\;\mathrm{Attentional}\;\mathrm{Bias}\;\mathrm{to}\;\mathrm{Negative}\;\mathrm{Information}\;=\;\mathrm{Number}\;\mathrm{of}\;\mathrm{correctly}\;\mathrm{identified}\;\mathrm{probes}\;\mathrm{in}\;\mathrm{the}\;\mathrm{locus}\;\mathrm{of}\;\mathrm{the}\;\mathrm{negative}\;\mathrm{talking}\;\mathrm{head}\;\mathrm{videos}/\mathrm{Total}\;\mathrm{number}\;\mathrm{of}\;\mathrm{correctly}\;\mathrm{identified}\;\mathrm{probes}$$

Scores on this index range from 0 to one, with higher values indicating relatively greater attention to the negative talking head videos compared to the benign talking head videos. A 0.5 score represents no attentional bias to either type of video.

The 24 dual videos were presented in a randomized order, with the constraint that dual videos concerning the same topic (e.g., health) were not presented again before dual videos concerning all five other topics were presented once. The 24 dual videos were organized into six blocks, each containing four dual videos, and participants were permitted a short break between each block.

#### Assessing the impact of emotional information on state anxiety

To assess the impact of exposing participants to the emotional information conveyed by the dual videos, state anxiety was assessed at baseline, and state anxiety was again assessed after each block of dual video presentation within the Talking Heads Attentional Bias Assessment Task. State anxiety change score was calculated for each participant by subtracting their baseline state anxiety score from their average state anxiety score following the presentation of each block of dual videos. Higher scores indicated greater elevation of state anxiety in response to the presented emotional information.

#### Experimental hardware

The Talking Heads Attentional Bias Assessment Task was presented on 22-inch widescreen monitors at a resolution of 1920 × 1080 pixels with a 60-Hz refresh rate. Participant responses were entered using a standard keyboard with a numeric keypad and a mouse.

#### Procedure

Upon arrival at the laboratory, participants read the information sheet and consent form. After providing written consent, they received instructions for the dual-probe attentional bias assessment task. Participants were instructed that, during the presentation of the video pairs, they should simply let their attention do whatever it naturally does and identify any probes they saw using the number pad. The complete participant instructions are available as supplementary material. Participants practiced identifying the probe stimuli that would be presented in the task, and they also learned how to respond to the state anxiety assessment items. Next, they completed a short practice session of the dual-probe task in which they were shown two dual videos, each comprising two benign video clips not used in the main task. Participants then completed the baseline STAI-S6 measure before commencing the dual-probe attentional bias assessment task, and repeated this after each of the six task blocks. Finally, participants answered demographic questions before being debriefed about the study’s purpose and thanked for their participation. In total, the experiment took approximately 60 min to complete. The study was approved by the University of Western Australia Human Research Ethics Office.

#### Availability of the talking heads attentional bias assessment task

To achieve the study’s aim of making the developed attentional bias assessment task readily available to other researchers, all materials used to create the Talking Heads Attentional Bias Assessment Task, including the full set of talking head video stimuli, which are automatically downloaded into a separate stimulus folder, along with the task itself and instructions for both users and participants, are available at http://www.ermcare.com/experimental-resources.html.^1^ The documentation includes step-by-step instructions, from downloading and running the task to R scripts for preparing the recorded data for analysis. It also covers important technical details, such as compatible hardware and the necessary information to replicate the administration process used in the current study. Additionally, the documentation provides guidance on computing different internal consistency measures (Kuder-Richardson 20 and split-half reliability) for the dual-probe task data. The study was not pre-registered.

## Results

All data were analyzed using R Statistical Software^2^ (v4.1.3; R Core Team, 2023). As mentioned, participants who failed to identify probes in at least 80% of dual-probe presentations were excluded. This resulted in the exclusion of 14 participants. Excluded and retained participants did not differ on demographics or trait and state anxiety.^3^ The remaining participants, on average, correctly identified probes in 93.26% (*SD* = 4.71%) of dual-probe presentations. The descriptive statistics of the main variables are reported in Table 1. Full descriptive statistics and complete correlation matrices are available in the Supplemental File.

**Table 1:** Descriptive statistics of the main study variables (*n* = 154)

	M	SD	Min	Max	Skew	Kurtosis
Index of Attentional Bias	0.48	0.13	0.08	0.84	– 0.92	2.02
Trait Anxiety Score	46.29	10.43	23	69	– 0.08	– 0.66
State Anxiety Change Score	4.83	12.03	– 34.89	53.25	0.71	1.87

First, to determine whether the Talking Heads Attentional Bias Assessment Task met the criterion for internal consistency (i.e., showing an internal reliability of 0.70 or higher), we assessed the internal consistency of the Index of Attentional Bias to Negative Information using three different internal consistency assessment methods, i.e., Kuder-Richardson 20, and split-half reliability. For the split-half reliability method, in addition to the commonly used odd/even split, which we used to maximize accessibility and facilitate replication, we employed a more robust split-half reliability method using random permutations (Parsons, 2021; Parsons et al., 2019). Unlike the single reliability estimate from an odd/even split, the permutation approach calculates reliability using multiple random halves to produce a reliability distribution. As demonstrated in recent methodological work, permutation-based split-half reliability provides a more accurate and less biased estimate of internal consistency for cognitive tasks based on trial-level data than single split approaches or traditional coefficients (Kahveci et al., 2025). We used the splithalf package (Parsons, 2021) to estimate reliability based on a random-permutation approach with 10,000 permutations. It should be noted that the trials used for internal reliability analyses were the exact same trials used to compute the Index of Attentional Bias to Negative Information. The internal consistency for the task, as measured by Kuder-Richardson 20 (KR-20), was excellent at 0.94. The odd/even split-half reliability yielded a coefficient of 0.89. For the split-half reliability using 10,000 random splits, the Spearman-Brown corrected reliability estimates for all conditions were 0.93 (95% CI = 0.92 to 0.95). These results indicate that the Talking Heads Attentional Bias Assessment Task demonstrated excellent reliability across different internal consistency assessment methods, thereby meeting the criterion for internal consistency.

Next, we tested whether the novel attentional bias assessment task demonstrated sensitivity to the well-established trait-anxiety-linked attentional bias effect. To test this, we computed a zero-order correlation between participants’ Index of Attentional Bias to Negative Information and their STAI-T scores. This analysis revealed a significant positive association between the Index of Attentional Bias to Negative Information and STAI-T scores, *r*(152) = 0.20, *p* = 0.013. This finding indicates that higher levels of trait anxiety were indeed associated with heightened attention to negative information relative to benign information, as indexed by this new Talking Heads Attentional Bias Assessment Task. Therefore, the new task met the criterion for the sensitivity to individual differences in trait anxiety.

**Fig. 3:**
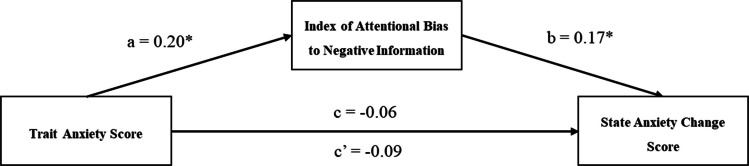
Diagram of mediation analysis of trait anxiety on state anxiety during the dual-probe task through attentional bias to negative information. Notes: *p <.05, **p <.01. Standardized regression coefficients are shown; c is the total effect of trait anxiety on state anxiety that does not consider the mediator, while c’ is the direct effect model with the mediator included

Next, we conducted an exploratory analysis to examine whether attentional bias to negative information predicts the magnitude of elevation in state anxiety from before to after information exposure and, moreover, to test the possibility that higher trait anxiety may indirectly predict elevation in state anxiety through attentional bias to negative information. This was tested using a mediation model in which the STAI-T score was the predictor, the State Anxiety Change score was the dependent variable, and the Index of Attentional Bias to Negative Information score was the proposed mediator. This mediation analysis was conducted using the PROCESS syntax (Hayes, 2009) with bias-corrected bootstrapping (10,000 resamples) to generate a 95% confidence interval. First, as shown in Fig. 3, trait anxiety significantly predicted higher attentional bias to negative information, which in turn predicted heightened elevation of state anxiety in response to emotional information. More importantly, the computed bootstrapped confidence interval for the indirect effect was above zero (0.002 to 0.084), indicating that trait anxiety indirectly predicted the impact of emotional information on state anxiety through attentional bias to negative information. Notably, the total effect of trait anxiety on state anxiety elevation was not significant, indicating an indirect-only mediation pattern. This result shows that the Talking Heads Attentional Bias Assessment Task provides a measure that mediates the relationship between trait and state anxiety.

## Discussion

The present study aimed to develop, validate, and make available to researchers an easy-to-implement attentional bias assessment task that demonstrates three key qualities: strong internal consistency, the use of ecologically valid stimuli conveying genuine issues that people may commonly engage with in real life, and sensitivity to the phenomenon of interest. The results confirmed that the newly developed Talking Heads Attentional Bias Assessment Task successfully demonstrated strong internal consistency and sensitivity to the phenomenon of interest. Regarding ecological validity, because we employed video-based stimuli that allowed the continuous presentation of information designed to reflect common topics people encounter in everyday life, it is reasonable to assume that the developed task has greater ecological validity compared to conventional single-probe tasks that rely on more impoverished stimuli, such as words or still images (Gerges et al., 2025; Schofield et al., 2015). The theoretical and methodological implications of the study are discussed in turn.

One key theoretical implication of the present findings is that they provide strong support for the well-documented trait anxiety-linked attentional bias effect using a task with improved ecological validity and internal consistency. Notably, the observed effect in the current study was demonstrated using stimuli that are complex, dynamic, and continuous – characteristics that more closely resemble real-world emotional information, compared to previous attentional bias assessment tasks that relied on impoverished stimuli of single words or still images (MacLeod et al., 2019). This reinforces the real-world applicability of the trait anxiety-linked attentional bias effect. Although the observed association between attentional bias and trait anxiety (*r* =.20) may appear modest in magnitude, it is important to contextualize this effect within the broader attentional bias literature. Converting this correlation to Cohen’s d (*d* =.41) yields an effect size that is directly comparable to the medium-sized effects reported in meta-analyses of traditional paradigms. For example, Bar-Haim et al. (2007) reported between-group threat-bias effects of *d* =.37 for the dot-probe task and *d* =.43 for emotional spatial cueing, with an overall between-group effect of *d* =.41. Thus, the magnitude of the present association is highly consistent with established findings in the field. Therefore, it is possible that the effect sizes observed in the present study reflect the true magnitude of the association between attentional bias to negative information and trait anxiety. Obviously, attentional bias represents only one of the numerous factors that contribute to anxiety, alongside many other biopsychosocial influences. However, it is also possible that the current task underestimates the strength of the association between attentional bias and trait anxiety. There are reasons to expect that future studies may observe stronger associations. For example, using stimuli that are personally relevant to participants may strengthen the effect. Importantly, the current task was intentionally designed to be broadly applicable rather than tailored to a specific group of individuals with particular sources of concern and anxiety. It is therefore expected that the magnitude of the association between vulnerability to anxiety and demonstrated attentional bias would increase as the personal relevance of the material increases, consistent with longstanding evidence that attentional bias effects are amplified when stimuli are disorder-congruent or personally salient rather than broadly negative (e.g., Pergamin-Hight et al., 2015; Dear et al., 2011).

The mediation analysis findings suggest that attentional bias to negative information may be a mechanism through which trait anxiety contributes to the elevation in state anxiety following exposure to emotional information, underscoring its role in heightened emotional reactivity among individuals with elevated trait anxiety. It is noteworthy that there was no significant direct correlation between trait anxiety and the elevation in state anxiety. However, the absence of a significant direct effect does not invalidate the interpretation of the mediation analysis, as contemporary mediation frameworks do not require a significant total or direct effect for a meaningful indirect pathway to be present. Nonetheless, it is useful to consider why a direct association may not have emerged in the present context. One possible explanation is that the current study was not designed to expose participants to an upcoming stressor, which may have limited the magnitude of changes in state anxiety. In contrast, stronger state anxiety responses are often observed in studies where participants anticipate an upcoming stressor, such as a mock job interview or a test, particularly when the presented stimuli are relevant to that situation (e.g., Grafton et al., 2023). Future research could therefore incorporate an anticipated stressor into the experimental design to examine the same mediation model.

An important methodological implication of the present study is that the newly developed attentional bias assessment task enables researchers to address questions that require acceptable to excellent levels of internal consistency in attentional bias measurement. In particular, reliable assessment of attentional bias is essential for three key areas of research. First, a major application is in personalized treatment approaches, where classifying individuals based on attentional bias is required (Bonett & Wright, 2015; Shamai-Leshem et al., 2023). Second, in investigations into mechanisms underlying psychological constructs, mediation analyses are required to examine how attentional bias contributes to relationships between different variables (Mazidi et al., 2026; Rodebaugh et al., 2016). Third, in cognitive bias modification studies, detecting changes in attentional bias over time is critical for evaluating the effectiveness of interventions aimed at modifying attentional bias (Zhang & Smith, 2020). The availability of a task that provides an internally consistent measure of attentional bias enhances the ability of researchers to examine attentional bias as an individual difference variable, a mediator of psychological outcomes, and a target in interventions aimed at reducing anxiety-related vulnerabilities. Although the internal consistency estimates observed in the present study are substantially higher than those typically reported for the conventional attentional probe tasks, such as the dot-probe task, no direct comparison between tasks was conducted within the current study. Future research directly comparing the Talking Heads task and the dot-probe on reliability, as well as on the strength of their associations with anxiety-related measures, would provide a stronger basis for evaluating their relative utility.

Another major methodological implication of the present study is that the developed task can be adapted by modifying the video content to present different categories of information, making it applicable across a wide range of research areas. For example, replacing the videos with content relevant to specific anxiety domains would allow the task to assess attentional bias in various forms of anxiety, such as health anxiety, social anxiety, climate anxiety, test anxiety, and prenatal anxiety. Beyond anxiety research, the task can also be modified to examine attentional bias in other psychological domains. For instance, a version could be developed for depression research to assess attentional bias toward positive versus depressogenic information, for addiction studies to investigate adaptive versus maladaptive attitudes toward alcohol use, or for eating disorder research to assess attentional bias toward adaptive versus maladaptive attitudes toward body appearance. Additionally, the probe presentation format can be modified to create an attentional bias modification version, enabling researchers to systematically manipulate or train attention toward or away from specific stimuli (Rudaizky et al., 2026). This adaptability applies not only to the Talking Heads Attentional Bias Assessment Task but also to any of its potential variations.

While the present study demonstrates the utility of the developed attentional bias assessment task, several limitations should be acknowledged. First, the sample consisted of undergraduate students, and participants were not selected based on their anxiety pathology, which may limit the generalizability of the findings to other participant groups, including those with clinical levels of anxiety. Future studies could address this limitation by applying the current methodology to individuals with clinical levels of anxiety. Second, the present study assessed only the internal consistency component of measurement reliability, which is a critical quality of an appropriate task and a major limitation of previous attentional bias assessment tasks. Future research should examine whether the task also meets other potentially desirable psychometric criteria, including test–retest reliability. It is important, however, to recognize that test–retest reliability should only be considered a desirable quality of a task if the target construct is expected to remain stable over time. If attentional bias is inherently variable rather than a fixed characteristic, a low test–retest reliability might not indicate a limitation of the task itself but instead reflect the true nature of the construct being assessed (Grafton et al., 2021; MacLeod et al., 2019). Therefore, when evaluating test–retest reliability, it is crucial to consider whether attentional bias is conceptualized as a stable trait or as a more dynamic and variable phenomenon. Third, because negative and benign information were always presented in paired contrast, the attentional bias index reflects relative attention to negative versus benign content. Although this does not affect the interpretation of the current results, which concern relative attentional prioritization, the present bias measure cannot distinguish whether this bias reflects vigilance toward negative information, avoidance of benign information, or a combination of both. Future adaptations of the task could address this question by pairing negative and positive content separately with neutral information, allowing distinct indices of vigilance and avoidance to be derived. Fourth, the present task does not distinguish between different components of attention, such as initial orienting versus sustained attentional maintenance. As a result, the observed attentional bias reflects overall attentional prioritization across the trial rather than isolating specific temporal components of attentional processing. Finally, although the developed task can be easily deployed without requiring specialized equipment, the current study relied on a single index of attentional bias based on participants’ accuracy in identifying probes. Future studies could incorporate complementary methodologies, such as eye-tracking, to test for converging evidence regarding attentional bias to negative versus benign information (Basanovic et al., 2022; Veerapa et al., 2020; Waechter et al., 2014).

Despite these limitations, the present study introduces a methodologically robust, ecologically valid, and readily available attentional bias assessment task that addresses some of the longstanding challenges in the field and offers valuable opportunities for both theoretical advancements and applied research. Its adaptability further enhances its potential for broader applications across other research domains, enabling future research to build on these findings and advance understanding and modification of attentional bias across various populations.

## Supplementary Information

Below is the link to the electronic supplementary material.

Supplementary file1 (PDF 106 kb)

## Data Availability

The de-identified data and data dictionary are openly available on the Open Science Framework at https://osf.io/qtzfy/files/osfstorage all materials used to create the Talking Heads Attentional Bias Assessment Task, along with the task itself and instructions for both users and participants, are available at http://www.ermcare.com/experimental-resources.html.

## Footnotes

1 We recognize that many researchers face limited assessment time constraints. To increase the task’s practical utility, we created a shorter version from the full-version that retains its psychometric properties. This version is described in the Supplementary Materials, along with a download link.

2 The de-identified data, data dictionary, and R scripts with descriptions for the analyses conducted in the current study are openly available on the Open Science Framework at https://osf.io/qtzfy/

3 All reported analyses were re-run for the full sample, and the pattern of results remained unchanged.

## Code availability

R scripts with descriptions for the analyses conducted in the current study are openly available on the Open Science Framework at https://osf.io/qtzfy/
