# Supplementary material for: The talking heads attentional bias assessment task: A readily available, reliable, and effective task for assessing attentional bias
Source: Behav Res Methods. 2026 May 22;58(6):171. doi: 10.3758/s13428-026-03045-6 (PMC13197368; doi:10.3758/s13428-026-03045-6)
Supplement: Supplementary file 1 — Supplementary file1 (PDF 106 kb) [file 13428_2026_3045_MOESM1_ESM.pdf]

The Talking Heads Attentional Bias Assessment Task: A Readily Available, Reliable, and Effective Task for Assessing Attentional Bias

Supplementary Materials

The Short Version of the Talking Heads Attentional Bias Assessment Task

We recognize that many researchers face constraints on assessment time. To increase the task's practical utility, we developed a shorter version of the task that includes half of the stimuli from the full version (i.e., 12 videos). In creating the short version of the task, we used a data-driven selection procedure with a repeated train–test split procedure to reduce overfitting and selection bias (Hastie et al., 2017). Across 2,000 random splits of the sample (70% training, 30% test), we first computed, within each training subsample, the correlation between trait anxiety and the attentional bias score derived from each of the 24 individual videos. The 12 videos showing the strongest associations with trait anxiety in the training subsample were selected and combined into a composite attentional bias index, which was then evaluated in the independent test subsample, consistent with recommended cross-validation practices for predictive modeling (Yarkoni & Westfall, 2017). We quantified the stability of video selection by calculating how frequently each video was retained across splits, following principles of stability selection and resampling-based inference (Meinshausen & Bühlmann, 2010). The final set of 12 videos consisted of those most consistently selected across repeated splits, thereby ensuring that the shortened task was based on videos demonstrating robust and replicable associations with trait anxiety rather than sample-specific variability.

For Review Only

To test whether the short version of the task also retained its psychometric properties and met the criteria for the required qualities of an appropriate attentional bias assessment task, we conducted the same analyses with the short version as those conducted with the full version of the task.

In terms of internal consistency, the split-half reliability using 10,000 random splits yielded a Spearman–Brown corrected reliability estimate of 0.89 (95% CI = 0.86 to 0.91) across conditions. With respect to sensitivity to individual differences in trait anxiety, the correlation between the attentional bias index derived from the short version of the task and trait anxiety scores was significant ($r = .295$, $p < .001$), and the correlation between the attentional bias index from the full task and the short version was $r = .92$, $p < .001$. These results indicate that the short version of the Talking Heads Attentional Bias Assessment Task also meets the required qualities.

The mediation model also showed the same pattern of results, with the attentional bias index significantly mediating the association between trait anxiety and state anxiety change scores, with the confidence interval of the indirect effect above zero (95% CI = 0.0034–0.1087).

The short version of the task could be downloaded from http://www.ermcare.com/experimental-resources.html.

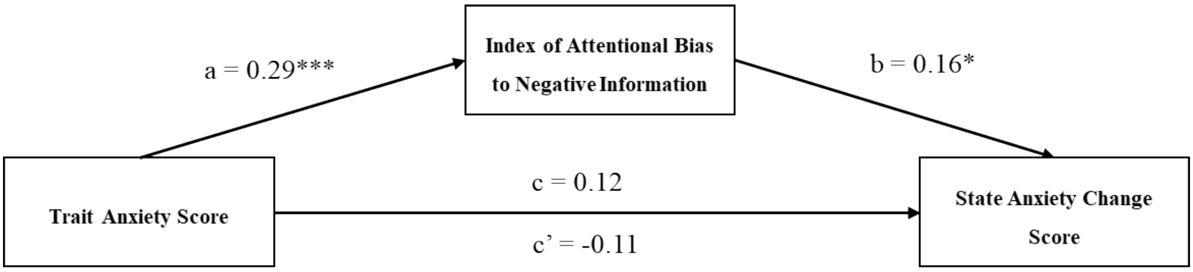

For Review Only

Fig. S1. Diagram of the mediation analysis of trait anxiety on state anxiety during the dual-probe task through attentional bias to negative information. Notes: * $p < .05$, ** $p < .01$. Standardized regression coefficients are shown; c represents the total effect of trait anxiety on state anxiety without considering the mediator, whereas c′ represents the direct effect with the mediator included.

References:

Hastie, T., Tibshirani, R. and Friedman, J. (2017) The Elements of Statistical Learning: Data Mining, Inference, and Prediction. 2nd Edition, Springer, Berlin.

Meinshausen, N., Bühlmann, P. (2010). Stability Selection, Journal of the Royal Statistical Society Series B: Statistical Methodology, Volume 72, Issue 4, Pages 417–473, https://doi.org/10.1111/j.1467-9868.2010.00740.x

Yarkoni, T., & Westfall, J. (2017). Choosing Prediction Over Explanation in Psychology: Lessons From Machine Learning. Perspectives on Psychological Science, 12(6), 1100-1122. https://doi.org/10.1177/1745691617693393

For Review Only

Table S1 shows the mean, standard deviation, and correlations between the assessed measures in the study.

Table S1

Means, standard deviations, and correlations between the study variables

Variable	*M*	*SD*	1	2	3	4	5	6	7	8	9	10	11
1. Index of Attentional Bias	0.48	0.13											
2. Trait Anxiety	46.29	10.43	.20*										
3. State Anxiety, assess 1	29.61	16.36	.15	.67***									
4. State Anxiety, assess 2	34.85	17.24	.14	.61***	.78***								
5. State Anxiety, assess 3	35.04	18.43	.22**	.60***	.76***	.90***							
6. State Anxiety, assess 4	34.18	19.11	.24**	.53***	.71***	.87***	.90***						
7. State Anxiety, assess 5	34.00	19.46	.24**	.50***	.71***	.82***	.86***	.92***					
8. State Anxiety, assess 6	33.92	20.08	.24**	.51***	.70***	.82***	.87***	.94***	.94**				
9. State Anxiety, assess 7	34.62	19.67	.24**	.50***	.68***	.82***	.85***	.87***	.88**	.91***			
10. State Anxiety Elevation	4.83	12.03	.15	-.06	-.22**	.31***	.38***	.48***	.47**	.50***	.49***		
11. Gender	73.85% Female		-.07	-.18*	-.13	-.14	-.12	-.17*	-.13	-.13	-.08	-.02	
12. Age	19.29	3.97	-.13	.05	.01	.05	.07	.04	.05	.06	.04	.07	.24**

Note. *M* and *SD* are used to represent mean and standard deviation, respectively. * indicates $p < .05$. ** indicates $p < .01$. *** indicates $p < .001$
For Gender, female was coded as 0 and male as 1

For Review Only
